# Mobile Technology Access and Use Among Adolescent Mothers in Lima, Peru: Mixed Methods Study

**DOI:** 10.2196/30240

**Published:** 2021-09-17

**Authors:** Elizabeth J Levey, Henry Onyeaka, Sophia M Bartles, Elena Sanchez Calderon, Sixto E Sanchez, Maria C Prom, Eden M Fesseha, Bizu Gelaye

**Affiliations:** 1 Chester M. Pierce Division of Global Psychiatry Massachusetts General Hospital Boston, MA United States; 2 Department of Psychiatry Harvard Medical School Boston, MA United States; 3 Gilling School of Global Public Health University of North Carolina Chapel Hill, NC United States; 4 AC PROESA Lima Peru; 5 Instituto de Investigaciòn Facultad de Medicina Humana Universidad de San Martìn de Porres Lima Peru; 6 Harvard College Cambridge, MA United States; 7 Department of Epidemiology Harvard T.H. Chan School of Public Health Boston, MA United States

**Keywords:** access to care, adolescent motherhood, LMICs, mobile phone, perinatal mental health, telehealth

## Abstract

**Background:**

Research shows promise for the use of mobile health interventions to improve access to care for mothers and infants. Although adolescent mothers in particular are comfortable with technology and often face barriers to accessing care, data on the use of digital interventions with young mothers are limited.

**Objective:**

This study aims to examine technology access and use behavior among adolescent mothers in Lima, Peru, to inform the development of technology-mediated perinatal interventions for high-risk mothers and infants in low- and middle-income countries and other areas with limited access to care.

**Methods:**

This mixed methods study consisted of a phone survey about technology access (N=29), focus group discussions with clinicians (N=25), and semistructured in-depth interviews with adolescent mothers (N=10) and their family members (N=8) in Lima.

**Results:**

All adolescent mothers surveyed had access to a smartphone, and nearly half had access to a computer or tablet. However, participants reported a number of obstacles to consistent smartphone access related to the financial precarity of their situations. Examples of this included difficulty affording phone services, using shared plans, and losing smartphones because of theft.

**Conclusions:**

These findings indicate that adolescent mothers are connected to technology, highlighting the potential scalability of technology-based health interventions for adolescent mothers in low- and middle-income countries while identifying barriers that need to be addressed.

## Introduction

### Background

According to the International Telecommunication Union’s 2016 report, 95% of the world lives in an area covered by a mobile cellular network and 85% is covered by cell phone signals [1,2]. Over the past few years, there has been significant growth in the use of mobile health (mHealth) technologies in low- and middle-income countries (LMICs), with infectious diseases and maternal health being the most frequent targets for these interventions [3,4]. mHealth interventions can be categorized by their purpose, including prevention and education, information sharing, and communication [5]. mHealth technologies hold promise for addressing challenges in health care access, delivery, and knowledge by better reaching individuals who may have difficulty accessing services [6].

Studies have identified maternal and child health as areas in which mHealth and other digital interventions hold significant promise for improving health outcomes in LMICs. A systematic review of the use of mHealth interventions in LMICs to address maternal health challenges found that mobile technologies had been used successfully in several areas, including data collection, decision support, and appointment reminders. Although these interventions showed promise for improving maternal health in LMICs, most of the studies were of low to moderate quality, indicating a need for more rigorous methods in this area [7]. Mothers in LMICs often do not receive the recommended number of antenatal or postnatal care contacts and experience gaps in knowledge and self-efficacy to provide care for their children at home [8]. mHealth interventions during this time can help improve access to information and care [8]. A recent systematic review focused on the use of mHealth educational interventions during the perinatal period and found that mothers who received these interventions had a significantly greater number of antenatal and postnatal care contacts [9]. A particularly successful example of an mHealth intervention that was scaled up in South Africa was the Mobile Alliance for Maternal Action project. This project connected mothers to the health care system by SMS text messaging, encouraged them to attend antenatal visits, and gave them information about pregnancy and childcare [10].

Maternal mental health is closely linked to maternal and child health outcomes [11-13]. For this reason, digital psychological interventions also hold promise for improving maternal and child health in resource-limited settings. For example, a digital intervention developed for low-income mothers living in rural areas of the United States was associated with increased maternal sensitivity and reduced maternal depression [14]. Despite the promise of technology to decrease disparities in access to care, research on digital interventions in LMICs has lagged behind high-income countries (HICs). However, in the past decade, researchers have begun to explore the use of technology-mediated mental health interventions in LMIC settings. In a systematic review of 19 telemental health intervention studies, the findings varied markedly based on how the intervention was delivered and the control condition used [15]. A recent systematic review and meta-analysis of 22 studies of digital psychological interventions in LMICs found that the interventions were moderately effective compared with usual care and concluded that digital interventions should be used in areas where there is inadequate access to in-person treatment [16]. Overall, this research indicates a rising trend in the use of mHealth and other digital interventions in LMICs, as well as a need for more empirical studies in this area [15].

LMICs account for 95% of adolescent births globally [17]. The adolescent pregnancy rate in LMICs is 48 births per 1000 girls (aged 15-19 years) per year, more than triple the rate in HICs (15 births per 1000 girls) [18]. Although mHealth interventions, specifically for adolescent mothers, are largely absent from the literature, the success of digital psychological interventions with adolescents suggests that it could be a useful tool to close the gap in access to care in this population. Digital psychological interventions have been demonstrated to be acceptable and feasible for adolescents [19,20]. The anonymity that internet-based interventions provide is reassuring to adolescents who are particularly sensitive to social stigma and have greater comfort with technology [21]. One systematic review of 22 randomized controlled trials of technology-mediated interventions for adolescents with depression or anxiety found improvements in depression and anxiety symptoms at the end of the intervention, but few studies conducted long-term follow-up [20].

Finally, mobile technology may also hold promise for measuring outcomes for parenting interventions, although there is limited research in this area in LMICs. Traditional methods of measuring parenting behavior, including dyad observation and retrospective questionnaires, can be resource intensive. To address these challenges, Fatori et al [22] tested the use of an electronic daily diary to measure the effects of a nursing home visiting program for adolescent mothers in Brazil. They found both high compliance to the electronic daily diary (84%) and a positive impact of the intervention on parental well-being and maternal parenting [22].

Although mHealth interventions hold potential to improve maternal health and adolescent parenting, LMIC settings face unique barriers to successful implementation. For the Mobile Alliance for Maternal Action project, described earlier, facilitators for this intervention’s scale-up included political will, stakeholders’ engagement, and adaptation to the South African context, whereas cost and financial sustainability were identified as barriers to scale-up [10]. Additional barriers identified to the scale-up and use of mHealth interventions in LMICs include lack of infrastructure or equipment, limited economic resources and literacy of users, privacy and confidentiality concerns, data costs, and network coverage and speed; only 7% of broadband subscriptions in LMICs have speeds of 10 megabits per second or higher [1,3].

### Objectives

In summary, research shows promise for the use of mHealth interventions to improve maternal and child health by increasing affordable access to care. However, although adolescent mothers, in particular, are more comfortable with technology and can have difficulty accessing hospital- or clinic-based care, data for the use of mHealth interventions with adolescents and in LMIC settings are limited. To address some of these gaps in the literature, this study examined technology access and use behavior among adolescent mothers in Lima, Peru, to inform the development of technology-mediated perinatal interventions for high-risk mothers and infants in LMICs and other areas with limited access to care. Peru is a middle-income country, with an adolescent fertility rate of 55 births per 1000 girls aged 15-19 years.

## Methods

### Study Setting

This study was conducted in Lima, Peru. Participants were recruited from 2 different clinical sites in Lima. Instituto Nacional Materno Perinatal (INMP) is the primary reference establishment for maternal and perinatal care operated by the Ministry of Health of the Peruvian Government. It serves low-income women who are publicly insured. Taller de Niños (TANI) is a nongovernmental organization that has been operating in Peru for more than 40 years, providing medical care and other services to infants and their families residing in the San Juan de Lurigancho District of Lima. With more than one million people, this district is Lima’s most populous district. Many of its residents have recently migrated from rural province areas.

### Participants and Procedures

#### Overview

This mixed methods study comprising quantitative phone surveys, qualitative focus groups, and in-depth individual interviews. The qualitative data were collected as part of a broader assessment of the needs and preferences of adolescent mothers and their families to inform the development of a perinatal intervention. This study focuses specifically on the findings related to the use of technology.

#### Quantitative

Quantitative data collection consisted of a 47-item phone survey that included questions about basic demographic information and technology access and use. The survey took approximately 15 minutes to administer. Pregnant and postpartum adolescents were recruited from patient records at INMP. Women between the ages of 14 and 19 who had given birth between May 1, 2019, and May 1, 2020, or were due to give birth before September 1, 2020, were eligible to participate. The goal was to survey 25 to 40 mothers to obtain a representative sample. Potential participants were contacted in May 2020. A total of 57 adolescents were contacted by phone and invited to participate in the survey; of these, 28 (49%) were unreachable by phone, whereas the remaining 29 (51%) were reached and agreed to participate.

#### Qualitative

Qualitative data collection consisted of 4 clinician focus groups (n=25), 10 in-depth interviews with adolescent mothers (n=10), and 8 in-depth interviews with other caregivers (n=8). The focus groups and interviewers were coconducted by 2 Spanish-speaking members of the research team. All focus groups and interviewers were audio recorded. One member of the research team asked questions, whereas the other took notes. Three focus groups were composed of clinicians from INMP, and one focus group was conducted with clinicians from TANI. After each focus group and interview, a member of the research team listened to the recording to review what had been discussed and consider how to explore topics of interest in greater depth until theoretical saturation was reached [23].

Participants in the clinician focus groups were recruited through the heads of the clinical services, who disseminated information about the study and worked with study staff to organize and schedule the focus groups. All clinicians who cared for adolescents during the perinatal period were eligible to participate in the study. The focus groups were conducted from July 1 to July 5, 2019. Each focus group was conducted in a private room at the clinical site and lasted for 90-120 minutes. The focus group discussion guide was designed to elicit clinicians’ perspectives on the needs of pregnant adolescents, adolescent mothers, and their families. This included educational, medical, physical, and emotional needs and ideas about how these needs could best be addressed. Focus group discussions were conducted before individual interviews, and major themes were identified and further explored in individual interviews.

Adolescent mothers who participated in the in-depth interviews were identified from birth records at INMP. Adolescents between the ages of 14 and 19 years who had given birth at INMP within the previous 15 months were eligible to participate. Potential participants were excluded if their infant had died, if they were not living in the Lima area, or if they did not speak Spanish. Interviews began on July 8, 2019, and were completed on February 22, 2020. Each interview was conducted in a private space at the participant’s home. The interviewer spent 4 hours at each home. Interviews were conducted in Spanish by a trained research assistant and lasted about 1.5 hours, and the rest of the time was spent conducting a more informal ethnographic observation of the living situation of the mother and infant. Similar to the focus groups, the recordings were reviewed after each interview to determine which topics needed to be explored in greater depth and identify when theoretical saturation was reached [23].

Participants were asked whether there was anyone helping them care for their infants who were available to be interviewed. This way, 8 other caregivers were identified—3 were fathers of the infants, 2 were mothers of the adolescent mothers, 1 was a mother-in-law, 1 was a grandmother, and 1 was a grandfather. In some cases, the adolescent mother and the other caregiver were interviewed sequentially during a single visit; in other cases, a separate in-person interview was scheduled.

The interview guide for adolescent mothers was designed and implemented by the researchers to understand the experiences of adolescent pregnancy and motherhood, identify their unmet needs, potential intervention targets, and potential barriers to and facilitators of acceptance of a perinatal intervention in this population. The interview guide for other caregivers was designed to elicit caregivers’ perceptions of the mother’s experience, as well as the caregivers’ own experiences and their relationships with their mothers. The research team made revisions to the interview guide after each interview to refine and deepen the questions. All participants provided informed consent. The institutional review boards of the INMP, Lima, Peru, and the Harvard T H Chan School of Public Health, Office of Human Research Administration, Boston approved all the procedures used in this study.

### Analysis

#### Quantitative Analysis

The survey data were stored and analyzed in Microsoft Excel [24]. Mean and SD statistics were calculated using this software.

#### Qualitative Analysis

Focus groups and interviews were transcribed verbatim by a Peruvian member of the research team and were then translated into English by a bilingual team member. The codebooks were developed in Spanish, and the transcripts were coded in Spanish. Once the coding was completed, the same coding was applied to the English translations so that the findings could be disseminated in English. A directed content analysis was used based on the goals of informing intervention adaptation [25]. Coders worked in 3 teams of 2, with one team coding focus groups, another team coding mother interviews, and a third team coding caregiver interviews. First, coders read the transcripts and developed initial codebooks over a series of meetings. Once coding began, the teams met after coding each transcript to discuss and revise the codebooks. The transcripts were then recoded using the revised codebook. Memoing was used throughout the process to generate new codes, refine definitions, and relate codes to each other [23]. Intercoder reliability was measured using the κ statistic [26].

Both inductive and deductive approaches were used to analyze data [23]. First, the manifest content was grouped thematically. Thematic groupings were then labeled, and these group labels were used to generate broad themes. These broad, overarching themes were divided into subthemes. Within each theme and subtheme, the researchers drew comparisons, looking for overlap and differences and newly emerging topics and patterns. The themes identified included technology access, barriers to access, attitudes toward technology use, problems associated with technology use, and expectations and desires related to technology. Responses were reviewed to identify each theme and subtheme. NVivo (QSR International) was used for data management [27]. Intercoder reliability was substantial (κ>0.6) [28,29]. For the focus groups, the intercoder reliability was κ=0.70. For adolescent mother interviews, the intercoder reliability was κ=0.67. For other caregiver interviews, the intercoder reliability was κ=0.79.

## Results

### Descriptive Data

A total of 29 participants completed the phone surveys (57 were contacted; 28 were unreachable). They were all between the ages of 14 and 19 years, with an average age of 17.8 years (SD 1.5). A total of 23 participants were pregnant, and 6 had given birth within the past year (Table 1).

**Table 1:** Demographic characteristics of phone survey participants (N=29).

Characteristic	Value
Age (years), mean (SD)	17.8 (1.5)
Pregnant, n (%)	23 (79)
Postpartum, n (%)	6 (21)

Participant demographics for the qualitative data are summarized in Tables 2-4. A total of 25 clinicians participated in the focus group. They were between the ages of 24 and 66 years, with an average age of 47.7 (SD 14.5) years. The clinical disciplines represented were physicians (obstetrician/gynecologists), nurses, psychologists, midwives, social workers, and community health workers. A majority of the clinicians were female; there were 3 male physicians and 1 male psychologist. The duration of their clinical experience ranged from less than a year to 43 years, with an average of 16.6 (SD 13.2) years (Table 2). Participants recruited from TANI were typically younger and had less clinical experience than those recruited from the INMP.

In-depth interviews were conducted with 10 adolescent mothers and 8 other caregivers. Adolescent mothers were an average age of 16.6 years (SD 1.6); the fathers of their infants were an average age of 21.2 years (SD 3.9). The age range of the infants was 2-14 months, with an average age of 6.8 (SD 4.2) months (Table 3). Of the 8 other caregivers interviewed, 3 (38%) were infants’ fathers, 2 (25%) were maternal grandmothers (mothers of the adolescents), 1 (13%) was a paternal grandmother, 1 (13%) was a maternal great-grandmother, and 1 (13%) was a maternal great-grandfather (Table 4).

**Table 2:** Demographic characteristics of focus group participants (N=25).

Characteristic	Value
Age (years), mean (SD)	47.7 (14.5)
Female, n (%)	21 (84)
Male, n (%)	4 (16)
Physicians, n (%)	5 (20)
Nurses, n (%)	5 (20)
Psychologists, n (%)	5 (20)
Midwives, n (%)	4 (16)
Social workers, n (%)	4 (16)
Community health workers, n (%)	2 (8)
Clinical experience (years), mean (SD)	16.6 (13.2)

**Table 3:** Demographic characteristics of adolescent mothers (N=10).

Characteristic	Value
Age of mothers (years), mean (SD)	16.6 (1.6)
Age of fathers (years), mean (SD)	21.2 (3.9)
Age of infants (months), mean (SD)	6.8 (4.2)
Living with family, n (%)	6 (60)
Living with partner, n (%)	4 (40)
Graduated high school, n (%)	3 (30)
Enrolled in high school, n (%)	3 (30)
Did not graduate and not currently enrolled in high school, n (%)	4 (40)

**Table 4:** Relationship of other caregivers to infants (N=8).

Relationship category	Participants, n (%)
Father	3 (38)
Maternal grandmother	2 (25)
Paternal grandmother	1 (13)
Maternal great-grandmother	1 (13)
Maternal great-grandfather	1 (13)

### Access to Technology

A phone survey of 29 pregnant and postpartum adolescents explored their access to technology (Table 5). All had access to a smartphone, and nearly half had access to a computer or tablet. However, there were a number of obstacles to consistent smartphone access. A total of 8 participants did not have their own phone but had only shared access to someone else’s phone and 9 had been without phone access at some time in the past 12 months for various reasons: 3 had their phones stolen, 3 had their phones break, and 3 lost their phones. A total of 21 participants used a prepaid phone plan, which was the most common way to access the internet, and 15 participants had wireless internet in their home. Difficulty in paying for phone or internet services was reported by 11 participants, and interruptions to electricity services were reported by 8 participants.

All participants reported using social media at least once per week, with 46% (13/28) reporting daily use, 36% (10/28) using it every other day, 14% (4/28) using it every three days, and 4% (1/28) reporting weekly use. Participants reported using the following 3 social media platforms: WhatsApp (28/28, 100%), Facebook (23/28, 82%), and Instagram (17/28, 61%). A total of 26 participants made video calls using their smartphones. Video streaming on their phones was done by 28 participants, but 54% (15/28) reported poor video quality.

**Table 5:** Mobile technology access among adolescent mothers (N=29).

Access type	Participants, n (%)
Computer or tablet	14 (48)
Smartphone	29 (100)
Shared smartphone	8 (28)
Lost smartphone access in previous 12 months	9 (31)
Prepaid phone plan	21 (72)
Wireless internet	15 (52)
Difficulty affording phone or internet service	11 (38)
Any social media use	29 (100)
Ability to make video calls	26 (90)

### Summary of Qualitative Findings

In the focus groups and in-depth interviews, participants spoke about access to technology, barriers or limitations to the use of technology, and how technology is used by adolescent mothers and their families, particularly as it relates to caring for themselves and their infants. Although many ideas were shared across participants, there were some key differences between the perspectives of clinicians and adolescent mothers regarding how adolescent mothers use technology. The other caregivers were heterogeneous—3 of them were fathers, and their experience with technology was similar to that of adolescent mothers. The remaining caregivers were older. As a group, they were less comfortable with technology, and some did not have smartphones.

### Barriers

A number of barriers were identified that limited access to technology, including theft, changing carriers, running out of data, and inability to afford any plan. Clinicians explained that many adolescents lived in dangerous areas where theft was common. For this reason, clinicians who did home visits were reluctant to bring their phones with them. They viewed this as a missed opportunity to use web-based tools to educate patients about pregnancy and childcare during their home visits. Phone theft also meant that adolescents were frequently without phones or using a different number. They also changed numbers when a particular carrier offered a special rate, or when they lost access to a shared phone. In addition, adolescent mothers reported that their access was limited because they were on a shared data plan, and they might run out of data, or the data could be cut off for periods of time if their family could not afford it. When this happened, they shifted to texting and other forms of communication that did not require data use. Alternatively, if they had no phone minutes and only internet access, they used apps such as WhatsApp, which uses internet rather than phone minutes. This was often the reason that they began using social media platforms to communicate. As one adolescent mother shared:

I only use WhatsApp because I don’t have a phone plan [now]. I talk to my dad and my grandmother, video calling with them.

As this example illustrates, adolescents with limited access to technology tended to limit communication to family and have less contact with those outside their immediate circle, including friends. In Peru and many LMICs, users are changed for placing calls and texting but not for receiving them. Thus, when they ran out of minutes, adolescents could receive calls, but they could not place them. For these reasons, it was difficult for clinicians to have reliable contact with their patients by phone.

Adolescents also preferred newer and more powerful technology; if they did not like their phones, they were less inclined to use them. As one adolescent mother explained, she did not use her phone as much since her previous phone was stolen because her new phone was smaller and less enjoyable to use: “I don’t feel like using this phone because it’s little, as opposed to mine, which was big.” Technology became a venue for comparison, including comparing devices and access. Adolescents who felt diminished by this comparison engaged less with technology.

### Technology for Education and Support

Clinicians described how technology can support adolescent mothers and improve their access to care. Some clinicians used texting to confirm appointments or alert patients that test results had become available. Community health workers said they gave their phone numbers to patients, and occasionally, they would use it to ask for help.

Some clinicians said that adolescents did not use technology for educational purposes, for example, to access information about their infants’ health or their own. However, a number of adolescent mothers and fathers reported that they used web-based searches to access information. One mother used web-based searches to learn about childbirth when she was pregnant. Others used the internet to learn more about introducing solid foods and how to manage common childhood ailments like diaper rash, as in the case of this father: “Most of all it was with the diaper rash and some test results that were sent to us online [that we used our smartphones].”

Adolescent mothers also used technology to help manage their daily tasks. This included coordinating with other members of their household and searching for answers to any number of practical questions related to cooking or home repair. They also used their smartphones to show their infants cartoons to quiet them or entertain them when they had to do household chores. They believed it was positive and stimulating for their infants: “That is what he likes because they do these mimics, thankfully, [he likes] the talking animals.”

### Social Connection

Adolescent mothers and clinicians both reported that adolescents used social media, but their attitudes toward this differed. Clinicians felt that social media use was not productive or useful in the service of the tasks that adolescent mothers needed to care for themselves and their infants. However, the accounts of adolescents painted a more complex picture. Social media did make it easier to stay connected with friends while being home with their babies, but the quality of the connection varied. Adolescent mothers reported that they were less socially engaged after having their babies. They did not go out and meet their friends regularly. They also reported that their social media use decreased but that social media still made it possible to stay connected in a more limited way. For example, they could post photos on social media and receive supportive comments from their friends. One adolescent found some classmates from childhood on Facebook and saw that some were also mothers. This was comforting to her as none of her friends had babies. However, she did not reach out to reconnect with these classmates:

There are several classmates who have had babies, but it's not that we talk; just that I see on Facebook that they do have babies. But they don't talk to me, we weren't very close, we were just acquaintances from class.

This participant found some comfort in finding others in similar situations on social media, but this did not necessarily lead to connection with them.

Those who were feeling alone because of estrangement from their families or significant conflict with them looked to social media for a sense of connection, but they found more disconnection and conflict. One adolescent described viewing pictures her friends posted and feeling like she was missing out on the social life that she had before becoming a mother. Another adolescent mother learned that her baby’s father had a new partner from pictures he posted on social media. For participants managing conflictual relationships, social media was a more acceptable way to engage because it felt more impersonal and, therefore, safer. For this reason, it could also be perceived as distancing and offending the recipient, as was the case with the mother of an adolescent, who responded to a Facebook message she received from her adolescent daughter:

How cruel that you mark your indifference against me, that you do not greet me, that you do as if you do not see me, you do not look up, you do not speak to me, you do not visit me, you do not know or ask how I am or anything.

Facebook greeting was experienced as slight when an in-person greeting was expected. In these instances, social media became another space in which the participants experienced relationship difficulties.

Adolescent participants also reported that they had difficulty setting boundaries around their social media communications. In the case of one participant who was estranged from her child’s father, the father discovered that she was in labor through Facebook and sent her a message. This was experienced as both an intrusion and a reminder of his absence. She was angry at him because of the way their relationship had ended, and contact from him at the moment she was in labor was overwhelming:

He’s the one that came here asking to meet his son because he found out that I had been at [the hospital]. The day that I gave birth he sent me a message on Facebook, but I didn’t know because I was giving birth, right? When they finally gave me my cellphone at midnight I saw his messages, and he wanted to come all the way from [neighboring country] that same day that my son was born. But I didn’t write back at all because I didn’t want him to meet my son. But he did meet him recently.

Although this participant ultimately allowed her child’s father to meet his son, her experience demonstrates that social media can make it easy to initiate contact and difficult to manage incoming contact. Another participant was awaiting a child support payment from her child’s father and received this message from the father’s new partner through social media:

Don’t worry, he’ll send you the monthly payment. Don’t get upset; don’t try to control him.

The participant experienced this as an unwanted intrusion. It was a communication about the missing payment, but it did not address the issue. Some adolescents found social media distracting when they were supposed to be doing other things, such as attending school. One participant avoided having data minutes on her phone so she could only use it when she was connected to internet at home:

No, if I reload it [with data minutes], it will distract me when I’m at school. Better not, no.

Adolescents found it difficult to restrict unwanted contacts on social media, and they also found it difficult to restrict their own use.

## Discussion

This study examined technology access, use patterns, and barriers among adolescent mothers in Peru. To the best of our knowledge, this is the first study to investigate the access and use of technology by adolescent mothers in LMICs. Despite the potential benefits of technology in promoting and improving access to care, the use of technology to support perinatal health among adolescent mothers remains relatively unexplored.

Overall, we found that adolescent mothers are connected to technology; all had smartphone access, and nearly half also had access to a laptop or tablet. These findings match the available data on adolescent technology access in HICs [30,31] and highlight the potential scalability of technology-based health interventions for adolescent mothers in LMICs. In addition, we found that all participants used social media at least once per week (13/28, 46% connected daily), and they had a range of experiences with it. Adolescent mothers reported difficulty in setting boundaries around their social media communications. These findings are consistent with past research, which has described the adverse impact of excessive social media use on adolescent mental health, well-being, and productivity [32-35]. Importantly, evidence indicates that these negative effects are stronger in girls than in boys [36]. Adolescent mothers and clinicians had different perspectives on adolescent technology use. They agreed that social media use could be problematic, but some of the helpful functions of technology were not visible to clinicians. Adolescents reported finding smart devices useful in searching for web-based health information, improving their health literacy, performing daily tasks, and supporting their care.

Similarly, some clinicians found these tools beneficial for disseminating test results, enhancing patient communication, and ensuring adequate follow-up care. However, they did not believe that adolescents used technology to attend to their own or their infant’s health; they saw it as purely a distraction. The clinician’s perspective may reflect a bias among clinicians to problematize patient behavior and focus on what they perceive patients to be doing wrong [37]. This may also indicate the need for guidance in adolescent technology use. Aschbrenner reported that adolescents preferred digital health interventions that included a professional moderator to manage peer-to-peer interactions [30].

Quantitative surveys were conducted during the pandemic, and qualitative interviews were conducted before the pandemic. We did not obtain qualitative data about the experience of the pandemic for adolescent mothers, but data on the impact of the pandemic on other vulnerable youth are relevant to this question [38]. School enrollment declined in many parts of the world [39], whereas violence and criminal behavior increased. However, in areas with adequate technology penetration, the increased availability of digital services improved access for some groups, including youth with chronic illnesses [40]. Adolescent mothers find it more difficult to leave their homes compared with other adolescents, and the expansion of services such as mHealth and remote learning may be particularly beneficial for them, which warrants further study.

Although a majority of participants had consistent access to technology, 3 major barriers were identified: (1) cost, (2) phone theft and loss, and (3) internet speed and signal strength. The cost of phones and data plans was a barrier; many adolescent mothers had difficulty affording smartphone services and thus relied on shared plans and sometimes shared devices. If they could no longer afford their plan, they would deactivate their device and use someone else’s. Although this allows them to maintain access, it means that they frequently change phone numbers and cannot be reached consistently by health care providers. This finding highlights the fact that adolescents can use mobile technology to access resources, but the health care system may have difficulty maintaining contact with them. This could limit the effectiveness of interventions that rely on consistent contact between adolescents and care providers, as adolescents in precarious circumstances may not be able to bear the responsibility for maintaining contact. They may be ambivalent about contact with the health care system, particularly if they feel criticized by providers. They may also be unable to manage and organize the information required to maintain contact. In addition, most phone plans in Peru are organized such that charges apply to calls and texts made but not to those received. Thus, each time adolescents change numbers, they must bear the expense of reconnecting.

Limited financial resources also meant that many adolescent mothers lived in neighbors with high rates of poverty where crime was endemic, and they experienced frequent device theft. This added to the need for shared devices, phone number changes, and other barriers to consistent contact. Owing to the crime, clinicians also feared bringing their devices on home visits to patients, limiting their ability to use technology as an educational tool.

Participants also reported that slow internet speed could interfere with the use of certain apps, including video streaming and videoconferencing, and rolling blackouts limited consistent access. Internet speed is typically slower and signal strength is weaker in LMICs, where 3G access exceeds 4G [41]. Areas where poverty is greatest and access to in-person care is most limited also face the greatest internet coverage gaps.

This study has several limitations. First, the sample size was small, with 29 adolescent mothers surveyed. Although the sample size for both the focus group discussions and the interviews was small, data analysis indicated that theoretical saturation was reached. In both phases of data collection, only Spanish speakers were recruited, which could limit generalizability and specifically applicability to indigenous communities in Peru. Second, participants were recruited from a single tertiary care women’s hospital in Lima, Peru. Adolescent mothers in other parts of Peru, or, for that matter, those receiving care at other clinical sites, were not included, and the data may not represent their experience. Third, there is the possibility of selection bias, as adolescents who chose to participate may be different from those who did not.

Our results indicate that adolescent mothers in Peru have access to digital technology, and some already use technology to help them care for their infants. These findings highlight the promise and potential of using scalable digital health interventions in LMIC settings to expand access to care and improve perinatal health outcomes. Of note, the pandemic is making it increasingly clear about the importance of telehealth in addressing health needs. However, barriers remain in the implementation of telehealth solutions in LMICs. A coordinated global approach centered around equity is needed to reduce these barriers by increasing personal device access, addressing limitations to maintaining access related to poverty, and growing internet connectivity. Subsidies are needed to increase technology access at the local level, and a coordinated global effort is needed to increase internet access as an important step toward reducing global health inequity.

## Abbreviations

HIC: high-income country
INMP: Instituto Nacional Materno Perinatal
LMIC: low- and middle-income country
mHealth: mobile health
TANI: Taller de Niños
